# Immune Checkpoint Inhibitor Associated Hepatotoxicity in Primary Liver Cancer Versus Other Cancers: A Systematic Review and Meta‐Analysis

**DOI:** 10.3389/fonc.2021.650292

**Published:** 2021-04-21

**Authors:** Jianyang Fu, Wang-Zhong Li, Nicole A. McGrath, Chunwei Walter Lai, Gagandeep Brar, Yan-Qun Xiang, Changqing Xie

**Affiliations:** ^1^ Thoracic and Gastrointestinal Malignancies Branch, Center for Cancer Research, National Cancer Institute, National Institutes of Health, Bethesda, MD, United States; ^2^ Department of Nasopharyngeal Carcinoma, Sun Yat-Sen University Cancer Center, Guangzhou, China; ^3^ Department of Medicine, Division of Gastroenterology and Hepatology, University of Maryland School of Medicine, Baltimore, MD, United States; ^4^ Sandra and Edward Meyer Cancer Center, Weill Cornell Medicine, New York, NY, United States

**Keywords:** immune checkpoint inhibitor, hepatotoxicity, primary liver cancer, PD-1, immune-related hepatitis

## Abstract

**Background:**

Overall risks of hepatotoxicity with immune checkpoint inhibitors (ICIs) have yet to be compared in primary liver cancers to other solid tumors.

**Methods:**

We reviewed data from the PubMed, Embase, and Scopus databases, and assessed the risk of hepatotoxicity associated with ICIs.

**Results:**

A total of 117 trials were eligible for the meta‐analysis, including 7 trials with primary liver cancers. The most common hepatotoxicity was ALT elevation (incidence of all grade 5.29%, 95% CI 4.52-6.20) and AST elevation (incidence of all grade 5.88%, 95% CI 4.96-6.97). The incidence of all grade ALT and AST elevation was 6.01% and 6.84% for anti-PD‐1 (95% CI 5.04-7.18/5.69-8.25) and 3.60% and 3.72% for anti-PD-L1 (95% CI 2.72-4.76/2.82-4.94; *p*< 0.001/p<0.001). The incidence of ≥ grade 3 ALT and AST elevation was 1.54% and 1.48% for anti-PD‐1 (95% CI 1.19-1.58/1.07-2.04) and 1.03% and 1.08% for anti-PD-L1 (95% CI 0.71-1.51/0.80-1.45; *p*= 0.002/p<0.001). The incidence of all grade ALT and AST elevation was 13.3% and 14.2% in primary liver cancers (95% CI 11.1-16.0 and 9.93-20.36) vs. 4.92% and 5.38% in other solid tumors (95% CI 4.21-5.76 and 4.52-5.76 in other solid tumors; *p* <0.001/*p*<0.001).

**Conclusion:**

Our study indicates that anti-PD-1 is associated with a higher risk of all‐ and high‐grade hepatotoxicity compared to anti-PD-L1, and primary liver cancers are associated with a higher risk of all‐ and high‐grade hepatotoxicity compared to other solid tumors.

## Introduction

Liver cancers are the fourth leading cause of cancer-related mortality worldwide (1, 2) with over 800,000 new primary liver cancer cases diagnosed around the world each year (3). Hepatocellular carcinoma (HCC) is one of the most aggressive liver cancers and accounts for 75-85% of these cases (1). Biliary tract carcinoma (BTC) originates in the intra- and extrahepatic biliary ductal system and is the second most common primary liver cancer after HCC. Surgical resection or liver transplantation are potential curative options for patients with early stage disease, however, the majority of patients present with advanced stage disease and are not candidates for curative therapies.

Immunotherapy has shown promising, effective and durable antitumor responses through immune mediated mechanisms by blocking internal immunosuppressive mechanism (e.g. CTLA-4 and PD-1/PD-L1 axis) and reactivating T cell-mediated host immune surveillance. Among these, immune checkpoint inhibitors (ICIs) have shown remarkable efficacy in the therapy of multiple cancers including HCC that has led to the regulatory approval of ICI in the first and second-line setting. Unfortunately, the antitumor effects of ICIs in BTC is much more limited and is still an ongoing active research field.

Although ICIs have achieved success as cancer therapies, their use comes with risks evidenced by toxic effects (treatment-related adverse events, TRAEs), including immune-related adverse events (IRAEs) caused by stimulation of the immune system (4–7). Those IRAEs are considered a consequence of T lymphocyte hyperactivation triggered by ICI introduction to attack auto-antigens located in the normal tissue. IRAEs lead to autoimmune-like manifestations in individual organs where the activated immune surveillance machinery attacks self-antigens on normal tissues. Hepatotoxicity derived from ICIs exhibits either a hepatocellular or cholestatic injury pattern with abnormal laboratory findings (8, 9). Clinically, hepatotoxicity can present with a range findings, from mild elevation of liver enzymes to hepatobiliary disorders like autoimmune hepatitis, cholangitis, jaundice and liver failure (10, 11). Currently, there are several meta-analyses that have evaluated hepatotoxicity from ICIs and found a general incidence of 2-30% (7, 8, 11–14).

Although there is overlapped antigenicity between normal liver tissue and tumors derived from the liver, it is unknown if ICI exposure can increase the incidence or severity of hepatotoxicity in patients with primary liver cancers in comparison to other cancer types. Therefore, we conducted a meta-analysis with the aim to compare liver-specific toxicities among patients with primary liver cancers versus other solid cancers.

## Methods

### Search Strategy and Study Selection

This study strictly followed the preferred reporting items for systematic reviews and meta‐analyses (PRISMA) guideline. A systematic search of the literature was conducted in PubMed, Web of Science and Embase to identify published clinical trials of ICIs that reported hepatotoxicity. The search was done using keywords anti-PD‐1, PD-1 inhibitor, anti-PD‐L1, PD-L1 inhibitor, nivolumab, pembrolizumab, avelumab, durvalumab and atezolizumab from June 1, 2008 through final research for updates on August 3, 2020. Phase I studies were excluded due to concerns about different dose ranges used. Studies eligible for this analysis must have met all of the following criteria: (1) prospective phase 2 or phase 3 human trials/cohorts in cancer therapy, (2) participants were treated with a single ICI agent to avoid cumulative hepatotoxicity from other agents, (3) reported tabulated data on any treatment-related hepatotoxicity, (4) sample size over 20, and (5) published in English. Meeting abstracts were excluded except for the phase 3 Checkmate 459 study that was included. When encountered with multiple publications from the same study population was identified, the one with the most recent, relevant and/or comprehensive hepatotoxicity data was included. The literature search, study selection, and data extraction were performed independently by 3 of the co-authors (F. J., W-Z. L and M.N.). The discrepancies were reviewed by another two investigators on the team (Y-Q. X and X.C) and resolved by consensus.

### Data Collection

A standard checklist was used for all studies including name of first author, year of publication, phase of the study, registration identity at clinicaltrial.gov, targeted cancer, ICI(s) involved, number of patients enrolled, number with liver metastases of enrolled patients, and number of patients recorded with hepatotoxicity in each trial. The hepatotoxicity in this analysis are all‐grades and high‐grade (≥grade 3) liver adverse events (AEs), which can be further classified into the elevation of alanine aminotransferase (ALT), aspartate aminotransferase(AST), blood bilirubin, blood alkaline phosphatase (ALP), γ‐glutamyl transpeptidase (GGT), immune related-hepatitis (ir-hepatitis), and hepatobiliary disorders (if separated from abovementioned items, including autoimmune hepatitis, acute hepatitis, liver failure, hepatic hemorrhage, transaminitis, elevated liver enzyme, cholecystitis, cholangitis, cholelithiasis, cholestasis, hepatocellular injury, drug-induced liver injury, jaundice, and hyperbilirubinemia).

### Statistical Analysis

Using the extracted data, we recorded the number of patients treated and the number of patients with hepatotoxicity reported. Meta-analysis of single incidence rates to calculate a pooled rate was performed using inverse variance method (15). Random effect models were fit using log transformation and were implemented to calculate a pooled incidence of each toxic effect and corresponding 95% confidence intervals (CIs). Continuity correction of 0.5 in studies with zero cell frequencies was applied in order to calculate individual study results with confidence limits and to conduct meta-analysis. Statistical heterogeneity was evaluated by the Cochran Q statistic and quantified with I^2^ statistics. A P value of less than 0.05 for *Chi*-square was regarded as the indicator of the presence of heterogeneity. I^2^ values of higher than 50% represented a high level of heterogeneity. To estimate the between-study variation in the meta-analysis, we also calculated the incidence rate of hepatotoxicity by study-level moderators, including the cancer type and drug type. Incidence differences between the different groups were compared with the Two-Proportions Z-Test.

All statistical analyses were performed by using R software (version 4.0.2, R Foundation for Statistical Computing) and the “meta” package.

## Results

### Eligible Studies and Characteristics

**Figure 1:**
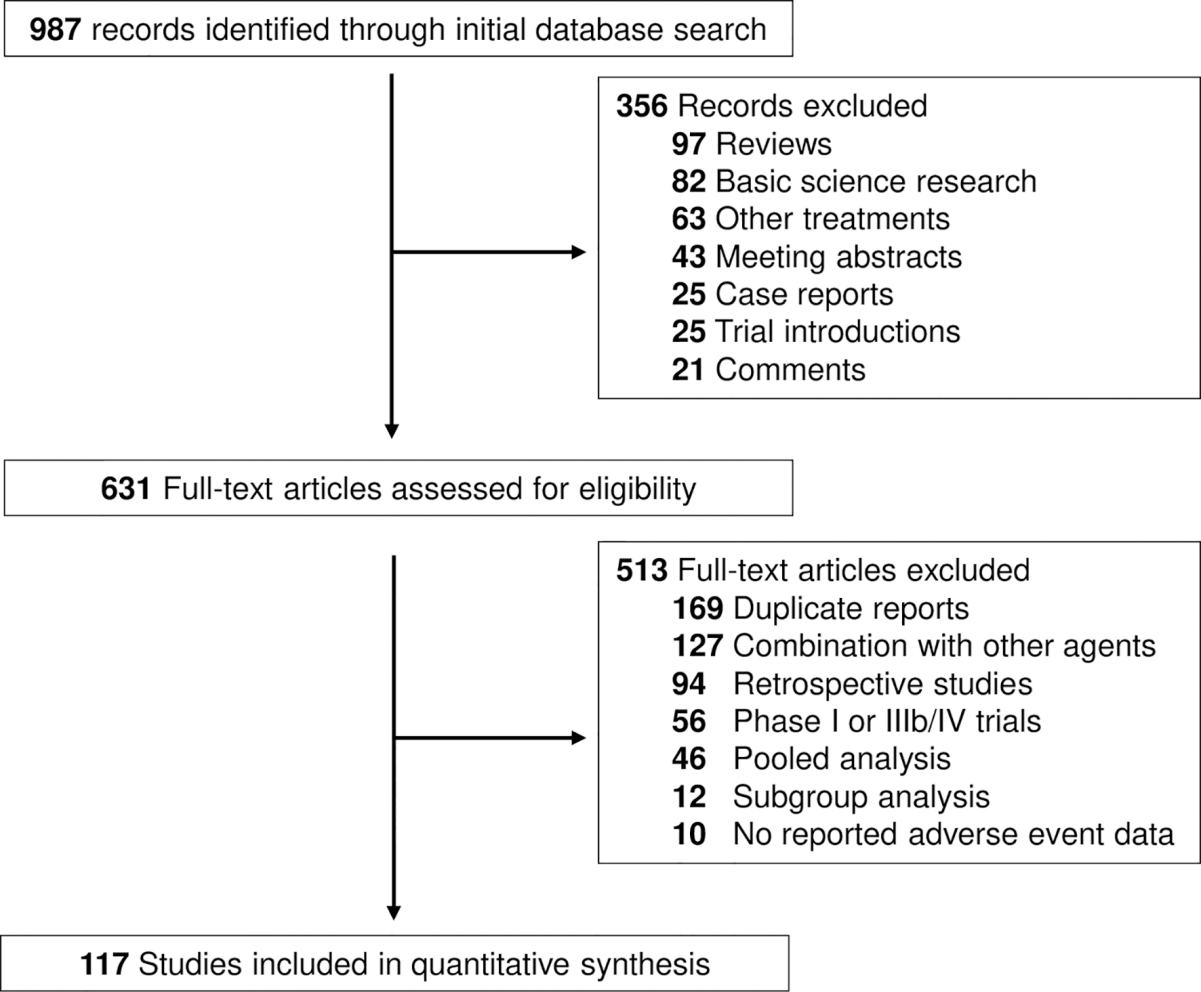
Flow diagram of study selection.

The search strategy revealed 987 potentially suitable records on ICIs from PubMed, Web of Science and Embase databases. The reasons for study exclusion are shown in **Figure 1**. Accordingly, a total of 117 clinical trials were considered eligible for the analysis (16–132). The trials involved the treatment of liver cancer (7 cohorts, n = 1362, 6 HCC and 1 BTC), lung cancer (27 cohorts, n = 7208), other gastrointestinal cancer (14 cohorts, n = 1798), genitourinary cancer (26 cohorts, n = 4963), melanoma (14 cohorts, n = 4169), head and neck cancer (10 cohorts, n = 1844), and other cancer types (15 cohorts, n = 994, including one cohort of thymic carcinoma, 2 cohorts of sarcoma, 2 cohorts of neuroendocrine tumor, 2 cohorts of mesothelioma, 3 cohorts of Merkle cell carcinoma and 5 cohorts of mixed solid tumors) (**Supplemental Table S1**). We categorized the regimens by ICI class using PD-1 inhibitor (88 cohorts; n = 16554 patients), and PD-L1 inhibitor (30 cohorts; n = 7113 patients). Specific PD-1 inhibitors included nivolumab (44 cohorts; n = 8368 patients) and pembrolizumab (44 cohorts; n = 7537 patients). Specific PD-L1 inhibitors included atezolizumab (14 cohorts, n = 4069), avelumab (5 cohorts, n = 743), and durvalumab (11 cohorts, n = 2301).

Some trials had not reported information of hepatotoxicity related to ICIs. **Supplemental Figure S1** shows the proportion and pattern of missing data from all trials included in this study. The most available reported hepatoxicity parameters are elevated ALT, AST, and hepatobiliary disorders, named transaminitis, acute liver injury, hepatocellular injury, and acute liver failure. The most common missed parameter was GGT that accounted over 60% missing data (**Supplemental Figure S1**). This may be due to the rare incidence of GGT change with ICI exposure that was absent from reports.

### Incidence of All-Grade Hepatotoxicity Across All Tumor Types

**Figure 2:**
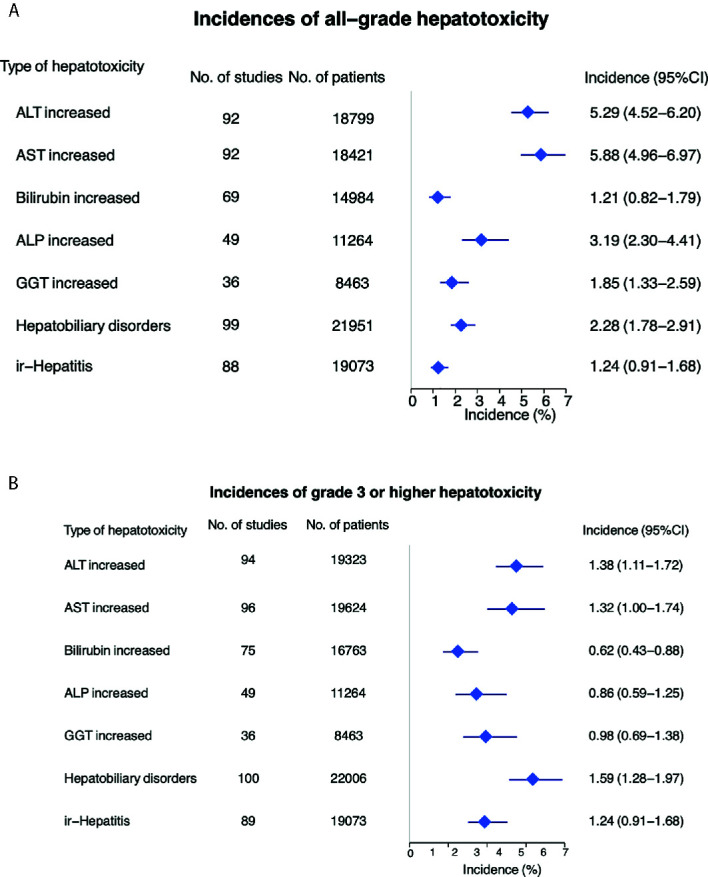
Incidence of all-grade **(A)** and high grade **(B)** hepatotoxicity across all tumor types.

All-grade hepatotoxicity parameters were reported as following (**Figure 2A** and **Table 1**): elevated ALT was reported in 92 studies (incidence 5.29%; 95% CI 4.52 - 6.20); elevated AST in 92 studies (incidence 5.88%; 95% CI 4.96 – 6.97); elevated bilirubin in 69 studies (incidence of 1.21%; 95% CI 0.82 – 1.79); elevated ALP in 49 studies (incidence 3.19%; 95% CI 2.30 – 4.41); elevated GGT in 36 studies (incidence of 1.85%; 95% CI 1.33 – 2.59); hepatobiliary disorder in 99 studies (incidence of 2.28%; 95% CI 1.78 – 2.91); and ir-hepatitis in 88 studies (incidence of 1.24%; 95% CI 0.91 – 1.68). Across all studies, the anti-PD‐1 subgroup showed statistically higher incidence of all-grade elevated ALT, AST, bilirubin and ALP, and ir-hepatitis in comparison with the anti-PD-L1 subgroup (*p*<0.05, **Supplemental Figure S2A** and **Table 2**), but not the incidence of elevated GGT and hepatobiliary disorders.

**Table 1:** Incidence of all grade and ≥grade 3 hepatotoxicity with ICIs (anti-PD-1/PD-L1).

Hepatotoxicity	All Grade (95% CI)	≥Grade 3 (95% CI)
ALT increased	5.29 (4.52−6.20)	1.38 (1.11−1.72)
AST increased	5.88 (4.96−6.97)	1.32 (1.00−1.74)
Bilirubin increased	1.21 (0.82−1.79)	0.62 (0.43−0.88)
ALP increased	3.19 (2.30−4.41)	0.86 (0.59−1.25)
GGT increased	1.85 (1.33−2.59)	0.98 (0.69−1.38)
Hepatobiliary disorders	2.28 (1.78−2.91)	1.59 (1.28−1.97)
Ir-hepatitis	1.24 (0.91−1.68)	0.97 (0.75−1.26)

ICIs, immune checkpoint inhibitors; ALT, alanine aminotransferase; AST, aspartate aminotransferase; ALP: alkaline phosphatase; GGT, γ‐glutamyl transpeptidase; Ir-hepatitis: immune related hepatitis.

**Table 2:** Incidence of hepatotoxicity secondary from anti-PD-1 vs. anti-PD-L1.

Hepatotoxicity	Grade	Anti-PD-1 (95% CI)	Anti-PD-L1 (95% CI)	*P* value
**ALT increased**	All	6.01 (5.04−7.18)	3.60 (2.72−4.76)	<0.001
	≥Grade 3	1.54 (1.19−1.58)	1.03 (0.71−1.51)	0.002
**AST increased**	All	6.84 (5.69−8.25)	3.72 (2.82−4.94)	<0.001
	≥Grade 3	1.48 (1.07−2.04)	1.08 (0.80−1.45)	<0.001
**Bilirubin increased**	All	1.36 (0.87−2.13)	1.04 (0.70−1.54)	<0.001
	≥Grade 3	0.63 (0.41−0.97)	0.63 (0.40−1.00)	0.294
**ALP increased**	All	3.89 (2.71−5.58)	1.64 (0.87−3.06)	<0.001
	≥Grade 3	0.92 (0.59−1.43)	0.74 (0.42−1.28)	0.112
**GGT increased**	All	1.66 (1.05−2.60)	2.16 (1.30−3.59)	0.405
	≥Grade 3	1.18 (0.87−1.61)	1.08 (0.69−1.68)	0.649
**Hepatobiliary disorders**	All	2.28 (1.70−3.07)	2.22 (1.38−2.57)	0.252
	≥Grade 3	1.60 (1.22−2.10)	1.55 (1.09−2.22)	0.460
**Ir-hepatitis**	All	1.17 (0.79−1.72)	1.37 (0.80−2.33)	0.043
	≥Grade 3	0.96 (0.71−1.29)	0.99 (0.60−1.65)	0.047

ALT, alanine aminotransferase; AST, aspartate aminotransferase; ALP, alkaline phosphatase; GGT, γ‐glutamyl transpeptidase; Ir-hepatitis, immune related hepatitis. Incidence differences between groups were tested with the Two-Proportions Z test.

### Incidence of High-Grade Hepatotoxicity Across All Tumor Types

The incidence of ≥ grade 3 hepatotoxicity was reported as follows (**Figure 2B** and **Table 1**): elevated ALT in 94 studies with an incidence of 1.38% (95% CI 1.11 – 1.72); elevated AST in 96 studies with an incidence of 1.32% (95% CI 1.00 – 1.74); elevated bilirubin in 75 studies with an incidence of 0.62% (95% CI 0.43 – 0.88); elevated ALP in 49 studies with an incidence of 0.86% (95% CI 0.59 – 1.25); elevated GGT in 36 studies with an incidence of 0.98% (95% CI 0.69 – 1.38); hepatobiliary disorders in 100 studies with an incidence of 1.59% (95% CI 1.28 – 1.97); and ir-hepatitis in 89 studies with an incidence of 0.97% (95% CI 0.75 – 1.26). Across all studies, the anti-PD‐1 subgroup showed statistically higher incidence of ≥ grade 3 elevation of ALT/AST and ir-hepatitis in comparison with the anti-PD-L1 subgroup (*p*<0.05, **Supplemental Figure S2B** and **Table 2**).

### Individual All-Grade Hepatoxicity in Primary Liver Cancers Compared to Other Cancer Types

**Figure 3:**
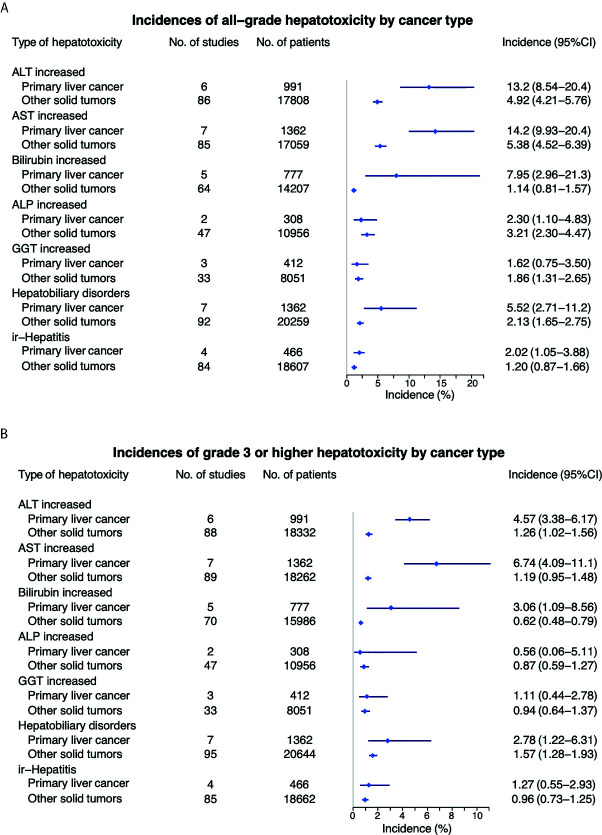
Comparison of incidence of all-grade **(A)** and high grade **(B)** hepatotoxicity between primary liver cancer vs other tumor types.

Overall incidence of all grade elevated ALT from anti-PD-1 or anti-PD-L1 in patients with primary liver cancer was 14.22% (95% CI 9.93 – 20.36); elevated AST 13.30% (95% CI 11.10 – 16.00); elevated bilirubin 7.95% (95% CI 2.96 – 21.33); elevated ALP 2.30% (95% CI 1.10 – 4.83); elevated GGT 1.62% (95% CI 0.75 – 3.50), non-specific liver toxicity was 11.7% (95% CI 9.74 – 14.00); and ir-hepatitis 2.02 (95% CI 1.05 – 3.88) (**Figure 3A** and **Supplemental Figure S3**). Interestingly, the incidence of all-grade elevated ALT, AST and bilirubin, and hepatobiliary disorders was statistically higher in primary liver cancer versus other cancer types (*p*<0.001, **Table 3**), but not the elevation of ALP/GGT and ir-hepatitis.

**Table 3:** Incidence of hepatotoxicity in primary liver cancers and other solid tumors treated with anti-PD-1/PD-L1.

Hepatotoxicity	Grade	Primary liver cancer (95% CI)	Other solid tumors (95% CI)	*P* value^*^
**ALT increased**	All	13.3 (11.1−16.0)	4.92 (4.21-5.76)	<0.001
	≥Grade 3	4.57 (3.38−6.17)	1.26 (1.02-1.56)	<0.001
**AST increased**	All	14.2 (9.93−20.36)	5.38 (4.52-5.76)	<0.001
	≥Grade 3	6.74 (4.09−11.11)	1.19 (0.95-1.48)	<0.001
**Bilirubin increased**	All	7.95 (2.96−21.33)	1.14 (0.81-1.57)	<0.001
	≥Grade 3	5.95 (4.07−8.71)	0.62 (0.48-0.79)	<0.001
**ALP increased**	All	2.30 (1.10−4.83)	3.21 (2.30-4.47)	0.401
	≥Grade 3	0.56 (0.08−3.95)	0.87 (0.59-1.27)	0.165
**GGT increased**	All	1.62 (0.75−3.50)	1.86 (1.31-2.65)	0.801
	≥Grade 3	1.11 (0.44−2.78)	0.94 (0.64-1.37)	0.384
**Hepatobiliary disorders**	All	11.7 (9.74−14.0)	2.13 (1.65-2.75)	<0.001
	≥Grade 3	6.25 (4.83−8.07)	1.57 (1.28-1.93)	<0.001
**Ir-hepatitis**	All	2.02 (1.05−3.88)	1.20 (0.87-1.66)	0.866
	≥Grade 3	1.27 (0.55−2.93)	0.96 (0.73-1.25)	0.768

ALT, alanine aminotransferase; AST, aspartate aminotransferase; ALP, alkaline phosphatase; GGT, γ‐glutamyl transpeptidase; Ir-hepatitis, immune related hepatitis. ^*^Incidence differences between groups were tested with the Two-Proportions Z test.

### Individual High-Grade Hepatoxicity in Primary Liver Cancers Compared to Other Cancer Types

The incidence of ≥ grade 3 hepatotoxicity from anti-PD-1 or anti-PD-L1 in patients with primary liver cancers was as follows: elevated ALT 6.74% (95% CI 4.09 – 11.11); elevated AST 4.57% (95% CI 3.38 – 6.17); elevated bilirubin 5.95% (95% CI 4.07 – 8.71); elevated ALP 0.56% (95% CI 0.08 – 3.95); elevated GGT 1.11% (95% CI 0.44 – 2.78), hepatobiliary disorder 11.7% (95% CI 9.74 – 14.00); and ir-hepatitis 1.27 (95% CI 0.55 – 2.93) (**Figure 3B** and **Supplemental Figure S4**). Furthermore, the incidence of ≥ grade 3 elevated ALT, AST, bilirubin, and hepatobiliary disorders were significantly higher in primary liver cancers versus other cancer types (*p*<0.001, **Table 3**).

## Discussion

In the present meta-analysis of all published phase 2 and 3 clinical trials of anti-PD-1 and anti-PD-L1 in patients with solid tumors, we reported the overall incidence of individual hepatotoxicity parameters secondary to treatment. There was an increased risk of all-grade and ≥ grade 3 ALT/AST elevation and ir-hepatitis when patients were treated with anti-PD-1 compared to anti-PD-L1. The overall incidence of all-grade and ≥ grade 3 elevated ALT, AST, bilirubin, and hepatobiliary disorders was increased in patients with primary liver cancer compared with other cancer types, treated with anti-PD-1/PD-L1, though incidence of reported ir-hepatitis was not significantly increased.

Hepatotoxicity triggered by ICI has been recognized as an important cause of morbidity and mortality and considered to be contributed by immune related mechanisms of action of ICIs. The elevation of ALT and AST is the earliest sign of liver injury. Hepatotoxicity can emerge days after ICI administration or be delayed by several months with a median onset ranging from 3 to 9 weeks (133). It commonly presents with isolated elevations of liver transaminases that can often subside after treatment discontinuation (133). Some severe cases are associated with other manifestations of liver dysfunction, e.g. coagulopathy, or even life-threatening liver failure. Previous meta-analyses have showed the incidence of all-grade liver toxicity induced by single agent of ICIs ranges from 2-37% (8, 12, 134–136) and this incidence increases when ICIs are administrated in combination with anti-CTLA-4 monoclonal antibodies or traditional chemotherapy (8, 136). In this study, we found that ALT and AST elevation were the most common presentation of all-grade treatment related hepatotoxicity with an incidence rate of 5.29% and 5.88%, respectively. There was a 1.24% incidence of reported all grade ir-hepatitis with high grade 0.97%. Other less common laboratory findings of liver toxicities included ALP elevation, hepatobiliary disorders, elevated bilirubin and GGT. The differences of reported incidence of hepatotoxicity from ICIs between this current study and previous results may be due to the substantial variations of terms used to report hepatotoxicity.

In addition, the anti-PD-1 treated patient population had higher incidence of all grade and ≥ grade 3 elevated ALT/AST and ir-hepatitis compared to anti-PD-L1. The causative mechanism is unclear. It has been proposed that the PD-L2 ligand may stimulate additional checkpoint signaling when anti-PD-L1 only targets PD-L1 versus anti-PD- blocks both PD-L1 and PD-L2 (135). Moreover, the anti-PD-1 executes an unselected T-Cell hyperactivation given generalized PD-1 expression on T cell membrane, that leads to potentially higher and more severe IRAEs than anti-PD-L1 (137). Furthermore, PD-1 expression is upregulated upon T cell activation and prevents the self-reactive and pathognomonic T cell activation (138). Therefore, the blockade of PD-1 by anti-PD-1 on dendric cells and other antigen-presenting cells could be relevant to worsen side effects than anti-PD-L1 (139). There are no head-to-head randomized clinical trials available to compare hepatotoxicity between anti-PD-1 and anti-PD-L1 inhibitors. Nevertheless, baseline liver dysfunction and primary or metastatic liver tumor burden in the trials may also contribute to these differences between trials using anti-PD-1 or anti-PD-L1.

Interestingly, in our study, there was a significantly higher incidence of elevated ALT/AST/bilirubin and hepatobiliary disorders in patients with primary liver cancers in comparison with other solid tumors. Although the majority of participants in the trials may or may not have abnormal liver function test results in the beginning with the background of liver disease, e.g. chronic viral hepatitis and cirrhosis, it is important to note that these underlying liver diseases may exacerbate/magnify the presentation of damaged liver functions after the administration of ICIs. Anti-PD-1/PD-L1 blocks the PD-1/PD-L1 pathway and activates T cells to target tumor antigens and kill tumors, but T cell activation may also be reactive against an antigen shared between normal tissue and tumors, that leads to normal organ injury (140). However, there is no consensus for the definition of drug induced ir-hepatitis related to ICI treatment in cancer. In our study, there was a significant percentage of missing information regarding ir-hepatitis. However, surrogate markers like elevated ALT/AST may be indication of ir-hepatitis and the incidence may be underestimated. This likely contributes to no difference seen between primary liver cancers and other solid tumors in incidence of ir-hepatitis.

The results of this analysis should be interpreted cautiously as there were several limitations to the study. First, there is publication bias given that the data was collected from published studies and only prevalent AEs were reported. For example, the incidence of > 5- 10% AEs were reported in the studies, dependent on the sample size of the trials. Since hepatotoxicity is not a common AE from ICIs, the information may not always be available for review. As a result, missing information can generate an overestimation of the incidence of hepatoxicity from ICIs. Second, potential co-founders were not reported in all publications. For instance, case numbers of liver metastasis for other solid tumors were missing. Metastatic liver lesions can result in hypoperfusion of normal liver tissues causing liver injury, which can be exacerbated by treatment caused by immune cell infiltration. Therefore, the incidence of liver toxicity directly contributed by ICIs in patients with solid tumors other than primary liver cancers could be inflated. Nevertheless, the hepatotoxicity derived from non-immune related factors is not able to be distinguished. Thirdly, the type and terms of reported hepatotoxicity were highly variable between trials, although every trial followed similar grading systems. Some studies reported hepatotoxicity as a whole labeled as one TRAEs or AE of interest, whereas others presented various laboratory liver function changes or clinical diagnoses like transaminitis and acute liver failure, or pathologic diagnoses like hepatocellular injury and liver injury. Moreover, there was significant variation or heterogeneity in the definition of hepatotoxicity across trials. Fourthly, the data was analyzed from published aggregated data and not from individual patients which is a common pitfall of meta-analysis data collection. It would be challenging to establish additional potential risk factors associated with the development of liver toxicity. Lastly, variations in the trial design, including eligibility criteria and phase of trial, can lead to selection bias. However, such a bias should not be significant because evaluation for hepatotoxicity was done with objective laboratory tests, rather than subjective AEs.

## Conclusion

This analysis found that primary liver cancers are associated with a higher risk of all‐ and high‐grade hepatotoxicity compared to other solid cancer types. Additionally, we found that anti-PD‐1 therapy results in a higher risk of hepatotoxicity compared to anti-PD-L1.

## Data Availability Statement

The original contributions presented in the study are included in the article/**Supplementary Material**. Further inquiries can be directed to the corresponding author.

## Author Contributions

Conceptualization Y-QX, CX. Methodology JF, W-ZL, Y-QX, CX. Software JF, W-ZL. Validation Y-QX, CX. Formal analysis JF, W-ZL, CX. Investigation JF, WZ-L, NM, Y-QX, CX. Resources JF, WL, NM, CX. Data curation JF, W-ZL, NM, CX. Writing—original draft preparation JF, NM, CX. Writing—review and editing JF, W-ZL, NM, GB, CL, Y-QX, CX. Visualization Y-QX, CX. Supervision Y-QX, CX. Project administration NM, CX. Funding acquisition Y-QX, CX. All authors contributed to the article and approved the submitted version.

## Funding

Y-QX is sponsored by National Natural Science Foundation of China (Program Grants 81572665). CX is supported by NIH/NCI/CCR Physician-Scientist Early Investigator Program (ZIA BC 011888).

## Conflict of Interest

The authors declare that the research was conducted in the absence of any commercial or financial relationships that could be construed as a potential conflict of interest.
