# Supplementary material for: Immune Checkpoint Inhibitor Associated Hepatotoxicity in Primary Liver Cancer Versus Other Cancers: A Systematic Review and Meta‐Analysis
Source: Front Oncol. 2021 Apr 21;11:650292. doi: 10.3389/fonc.2021.650292 (PMC8097087; doi:10.3389/fonc.2021.650292)
Supplement: Supplementary file 1 [file Presentation_1.pptx]

## Slide 1
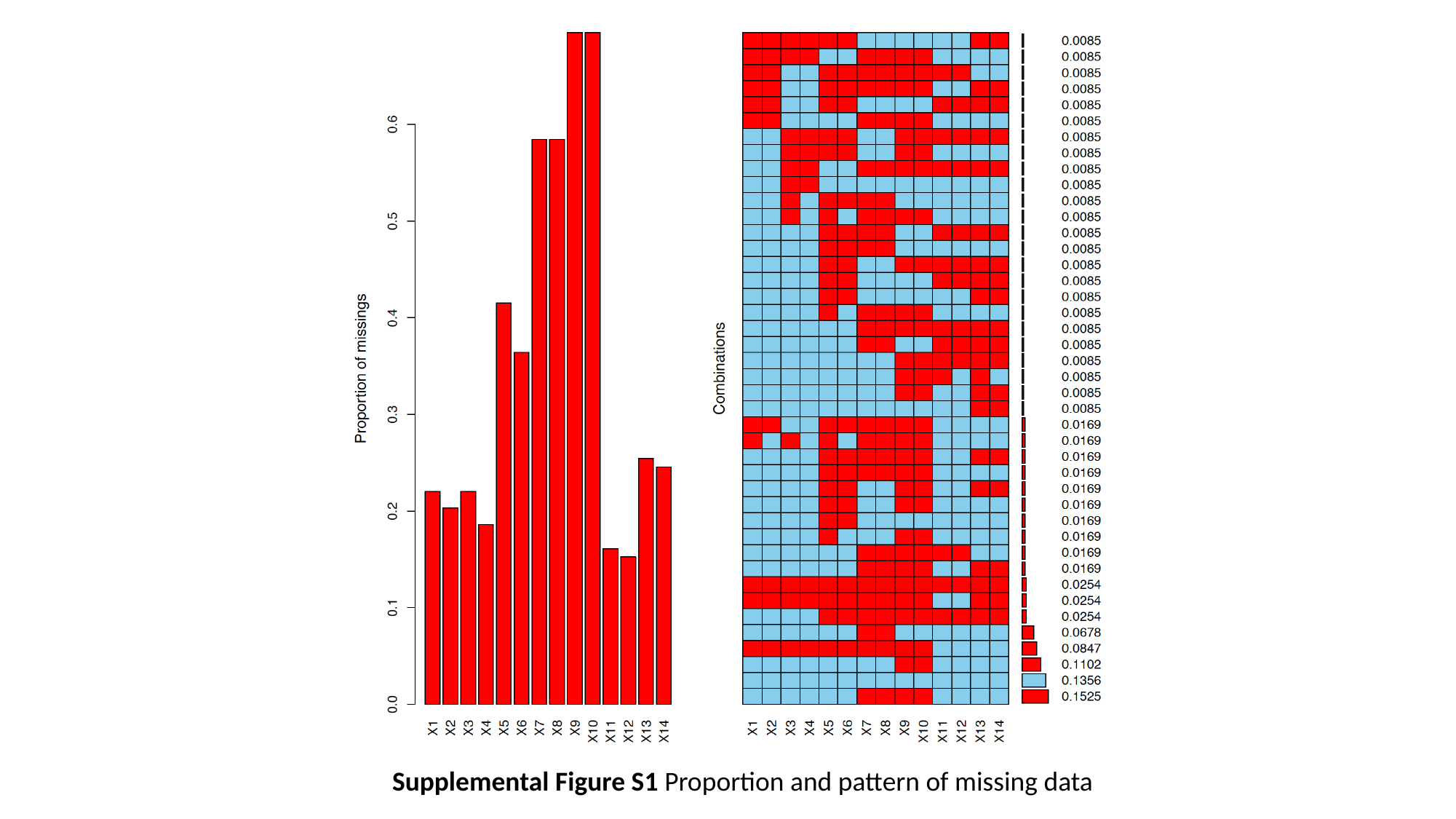

Supplemental Figure S1 Proportion and pattern of missing data

## Slide 2
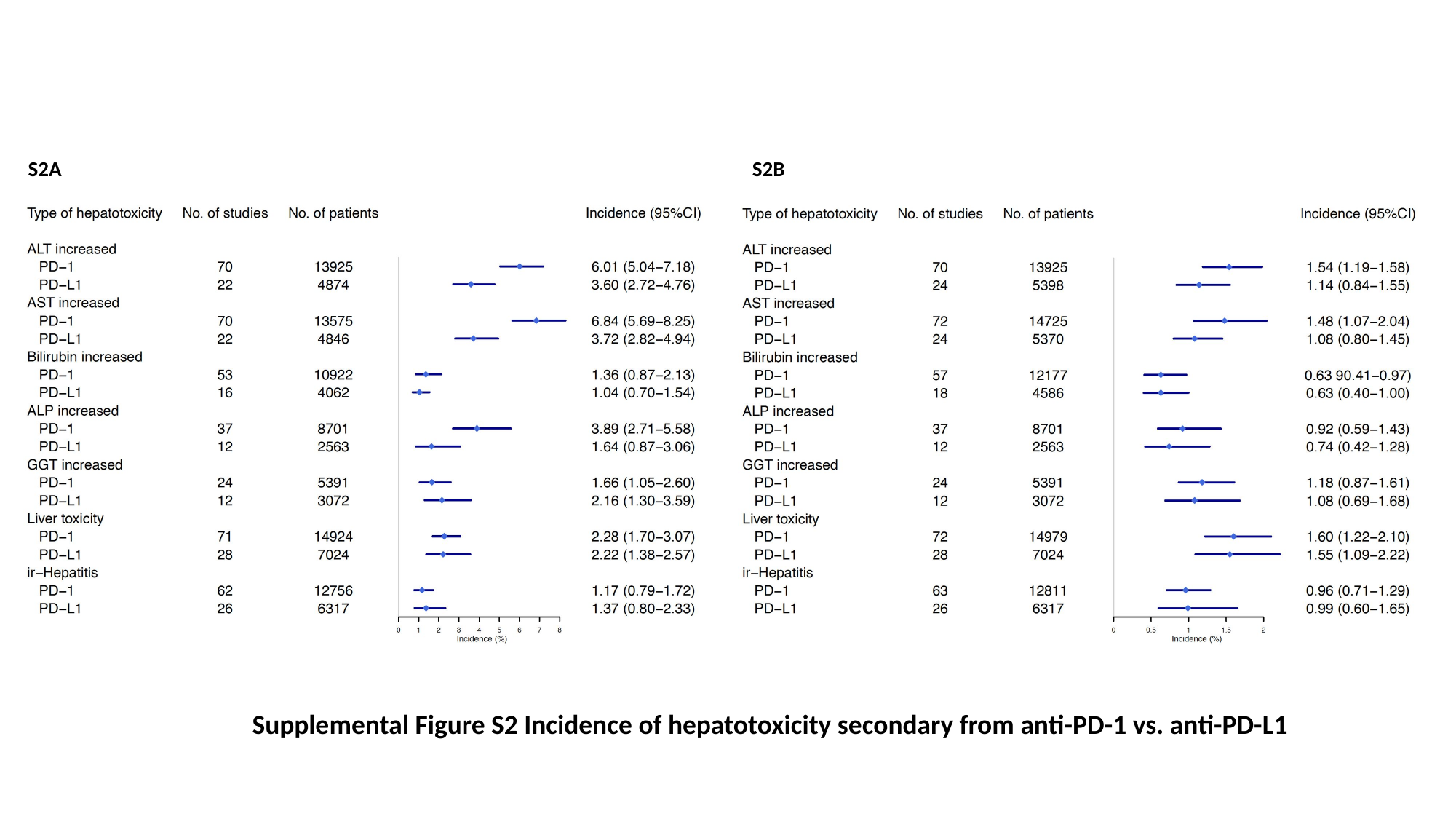

S2A
S2B
Supplemental Figure S2 Incidence of hepatotoxicity secondary from anti-PD-1 vs. anti-PD-L1

## Slide 3
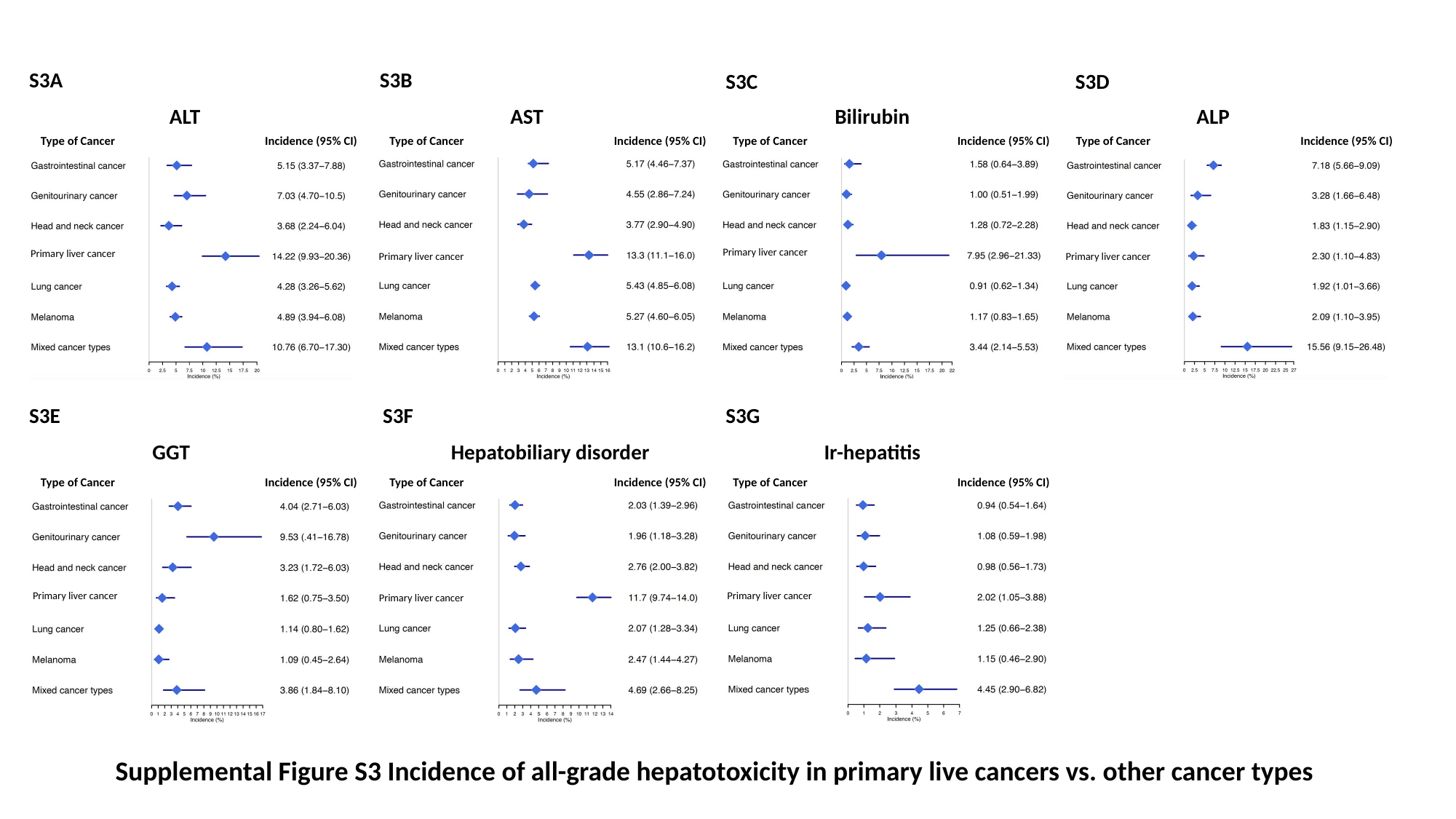

S3A
S3B
S3C
S3D
ALT
AST
Bilirubin
ALP
Type of Cancer Incidence (95% CI)
Type of Cancer Incidence (95% CI)
Type of Cancer Incidence (95% CI)
Type of Cancer Incidence (95% CI)
Primary liver cancer
Primary liver cancer
Primary liver cancer
Primary liver cancer
Type of Cancer Incidence (95% CI)
Type of Cancer Incidence (95% CI)
Type of Cancer Incidence (95% CI)
Primary liver cancer
Primary liver cancer
Primary liver cancer
S3E
S3F
S3G
GGT
Hepatobiliary disorder
Ir-hepatitis
Supplemental Figure S3 Incidence of all-grade hepatotoxicity in primary live cancers vs. other cancer types

## Slide 4
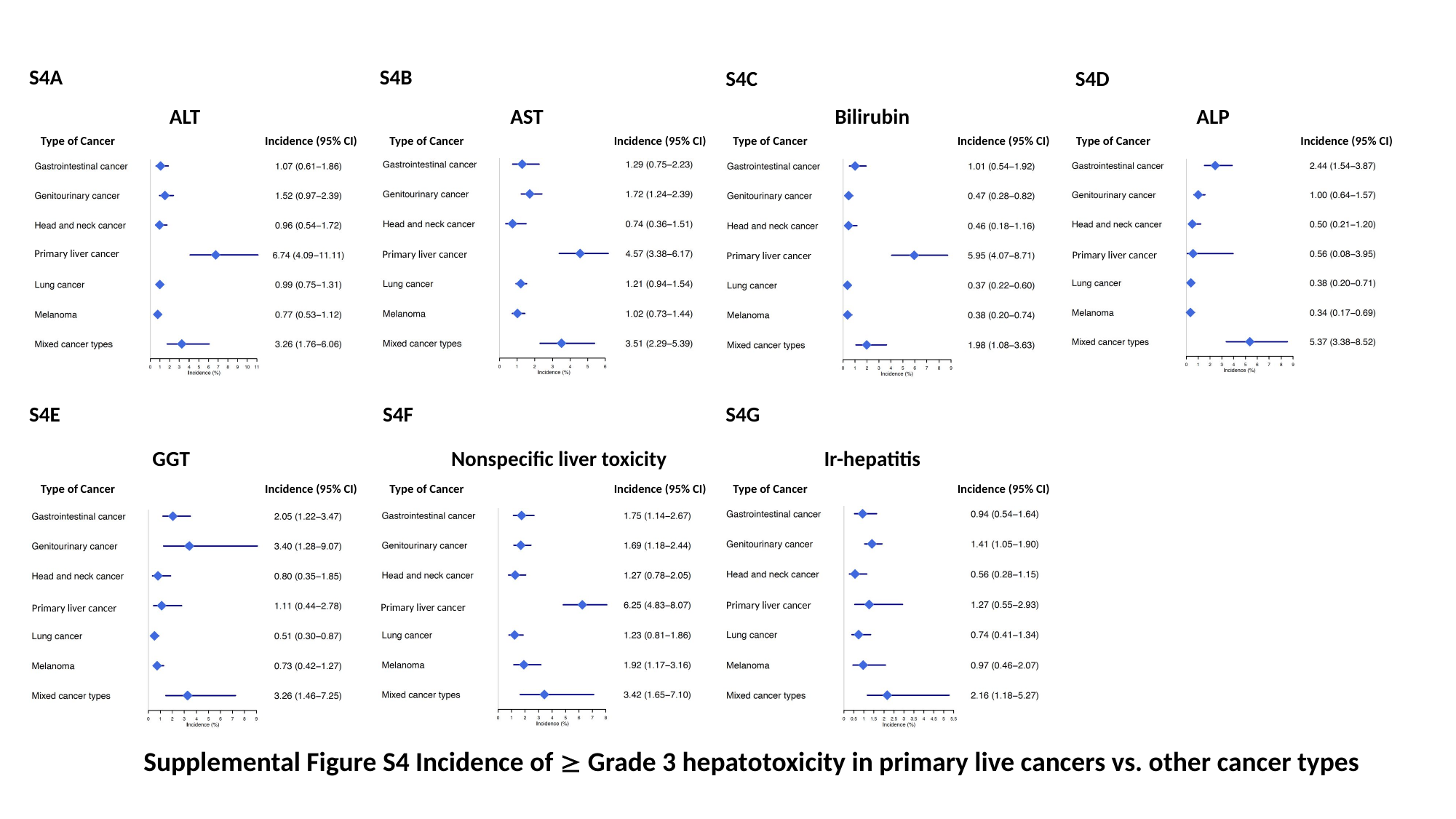

S4A
S4B
S4C
S4D
ALT
AST
Bilirubin
ALP
Type of Cancer Incidence (95% CI)
Type of Cancer Incidence (95% CI)
Type of Cancer Incidence (95% CI)
Type of Cancer Incidence (95% CI)
Primary liver cancer
Primary liver cancer
Primary liver cancer
Primary liver cancer
S4E
S4F
S4G
GGT
Nonspecific liver toxicity
Ir-hepatitis
Type of Cancer Incidence (95% CI)
Type of Cancer Incidence (95% CI)
Type of Cancer Incidence (95% CI)
Primary liver cancer
Primary liver cancer
Primary liver cancer
Supplemental Figure S4 Incidence of  Grade 3 hepatotoxicity in primary live cancers vs. other cancer types
