# Supplementary material for: Immune Checkpoint Inhibitor Associated Hepatotoxicity in Primary Liver Cancer Versus Other Cancers: A Systematic Review and Meta‐Analysis
Source: Front Oncol. 2021 Apr 21;11:650292. doi: 10.3389/fonc.2021.650292 (PMC8097087; doi:10.3389/fonc.2021.650292)
Supplement: Supplementary file 2 [file Table_1.docx]

**Supplemental Table 1 List of the Studies Included in This Meta-Analysis**

| **Journal** | **First author and publication year** | **Publication title** | **Drug type** | **Drug** | **Cancer type** | **Cancer location** | **Patient number** |
| --- | --- | --- | --- | --- | --- | --- | --- |
| The Lancet Oncology | Garassino 2018 | Durvalumab as third-line or later treatment for advanced non-small-cell lung cancer (ATLANTIC): an open-label, single-arm, phase 2 study | PDL1 | Durvalumab | Lung cancer | NSCLC | 444 |
| Journal of Clinical Oncology | Raj 2020 | PD-1 blockade in advanced adrenocortical carcinoma | PD1 | Pembrolizumab | Genitourinary cancer | Adrenocortical Carcinoma | 39 |
| Lancet Oncology | Morris 2017 | Nivolumab for previously treated unresectable metastatic al cancer (NCI9673): a multicentre, single-arm, phase 2 study | PD1 | Nivolumab | Mixed cancer types | Anal cancer | 37 |
| Lancet Oncology | Sharma 2016 | Nivolumab monotherapy in recurrent metastatic urothelial carcinoma (CheckMate 032): a multicentre, open-label, two-stage, multi-arm, phase 1/2 trial | PD1 | Nivolumab | Genitourinary cancer | Bladder cancer | 78 |
| Lancet Oncology | Sharma 2017 | Nivolumab in metastatic urothelial carcinoma after platinum therapy (CheckMate 275): a multicentre, single-arm, phase 2 trial | PD1 | Nivolumab | Genitourinary cancer | Bladder cancer | 270 |
| Nat Med | Voorwerk 2019 | Immune induction strategies in metastatic triple-negative breast cancer to enhance the sensitivity to PD-1 blockade: the TONIC trial | PD1 | Nivolumab | Genitourinary cancer | breast cancer | 68 |
| Annals of Oncology | Adams 2019_1 | Pembrolizumab monotherapy for previously untreated, PD-L1-positive, metastatic triple-negative breast cancer: cohort B of the phase II KEYNOTE-086 study | PD1 | Pembrolizumab | Genitourinary cancer | breast cancer | 84 |
| Annals of Oncology | Adams 2019_2 | Pembrolizumab monotherapy for previously treated metastatic triple-negative breast cancer: cohort A of the phase II KEYNOTE-086 study | PD1 | Pembrolizumab | Genitourinary cancer | breast cancer | 170 |
| JAMA oncology | Kim 2020 | A Phase 2 Multi-institutiol Study of Nivolumab for Patients With Advanced Refractory Biliary Tract Cancer | PD1 | Nivolumab | Hepatocellular carcinoma | BTC | 54 |
| Journal of Clinical Oncology | Chung 2019 | Efficacy and safety of pembrolizumab in previously treated advanced cervical cancer: Results from the phase II KEYNOTE-158 study | PD1 | Pembrolizumab | Genitourinary cancer | cervical cancer | 98 |
| Lancet Oncol | Overman 2017 | Nivolumab in patients with metastatic D mismatch repair-deficient or microsatellite instability-high colorectal cancer (CheckMate 142): an open-label, multicentre, phase 2 study | PD1 | Nivolumab | Gastrointestinal cancer | Colorectal cancer | 74 |
| The Lancet Oncology | Eng 2019 | Atezolizumab with or without cobimetinib versus regorafenib in previously treated metastatic colorectal cancer (IMblaze370): a multicentre, open-label, phase 3, randomised, controlled trial | PDL1 | Atezolimumab | Gastrointestinal cancer | Colorectal cancer | 90 |
| Cancer research and treatment | Kim 2020 | A Phase II Study of Avelumab Monotherapy in Patients with Mismatch Repair-Deficient/Microsatellite Instability-High or POLE-Mutated Metastatic or Unresectable Colorectal Cancer | PDL1 | Avelumab | Gastrointestinal cancer | Colorectal cancer | 44 |
| N Engl J Med | Le 2015 | PD-1 Blockade in Tumors with Mismatch-Repair Deficiency | PD1 | Pembrolizumab | Gastrointestinal cancer | Colorectal and others | 41 |
| Journal of Clinical Oncology | Azad 2020 | Nivolumab Is Effective in Mismatch Repair-Deficient Noncolorectal Cancers: Results From Arm Z1D-A Subprotocol of the NCI-MATCH (EAY131) Study | PD1 | Nivolumab | Mixed cancer types | dMSS/non CRC | 42 |
| Obstetrical & Gynecological Survey | Ott 2018 | Safety and Antitumor Activity of Pembrolizumab in Advanced Programmed Death Ligand 1-Positive Endometrial Cancer: Results From the KEYNOTE-028 Study | PD1 | Pembrolizumab | Genitourinary cancer | Endometrial Cancer | 24 |
| Journal of Clinical Oncology | Konstantinopoulos 2019 | Phase II Study of Avelumab in Patients With Mismatch Repair Deficient and Mismatch Repair Proficient Recurrent/Persistent Endometrial Cancer | PDL1 | Avelumab | Genitourinary cancer | Endometrial cancer | 31 |
| The Lancet Oncology | Kato 2019 | Nivolumab versus chemotherapy in patients with advanced oesophageal squamous cell carcinoma refractory or intolerant to previous chemotherapy (ATTRACTION-3): a multicentre, randomised, open-label, phase 3 trial | PD1 | Nivolumab | Gastrointestinal cancer | esophageal cancer | 209 |
| Lancet Oncol | Kudo 2017 | Nivolumab treatment for oesophageal squamous-cell carcinoma: an open-label, multicentre, phase 2 trial | PD1 | Nivolumab | Gastrointestinal cancer | esophageal cancer | 65 |
| JAMA Oncol | Shah 2019 | Efficacy and Safety of Pembrolizumab for Heavily Pretreated Patients With Advanced, Metastatic Adenocarcinoma or Squamous Cell Carcinoma of the Esophagus: The Phase 2 KEYNOTE-180 Study | PD1 | Pembrolizumab | Gastrointestinal cancer | esophageal cancer | 121 |
| Lancet | Shitara 2018 | Pembrolizumab versus paclitaxel for previously treated, advanced gastric or gastro-oesophageal junction cancer (KEYNOTE-061): a randomised, open-label, controlled, phase 3 trial | PD1 | Pembrolizumab | Gastrointestinal cancer | gastric or GE cancer | 294 |
| Lancet | Kang 2017 | Nivolumab in patients with advanced gastric or gastro-oesophageal junction cancer refractory to, or intolerant of, at least two previous chemotherapy regimens (ONO-4538-12, ATTRACTION-2): a randomised, double-blind, placebo-controlled, phase 3 trial | PD1 | Nivolumab | Gastrointestinal cancer | gastroesophageal cancer | 330 |
| Clinical Cancer Research | Kelly 2020 | Safety and efficacy of durvalumab and tremelimumab alone or in combination in patients with advanced gastric and gastroesophageal junction adenocarcinoma | PDL1 | Durvalumab | Gastrointestinal cancer | gastroespheal cancer | 24 |
| Lancet | El-Khoueiry 2017 | Nivolumab in patients with advanced hepatocellular carcinoma (CheckMate 040): an open-label, non-comparative, phase 1/2 dose escalation and expansion trial | PD1 | Nivolumab | Hepatocellular carcinoma | HCC | 214 |
| Journal of Clinical Oncology | Yau 2019 | CheckMate 40: Nivolumab in patients with advanced hepatocellular carcinoma and child-pugh status B | PD1 | Nivolumab | Hepatocellular carcinoma | HCC | 311 |
| ESMO | Yau T 2020 | CheckMate 459: A Randomized, Multi-Center Phase 3 Study of Nivolumab vs Sorafenib as First-Line Treatment in Patients With Advanced Hepatocellular Carcinoma | PD1 | Nivolumab | Hepatocellular carcinoma | HCC | 371 |
| Cancer | Feun 2019 | Phase 2 study of pembrolizumab and circulating biomarkers to predict anticancer response in advanced, unresectable hepatocellular carcinoma | PD1 | Pembrolizumab | Hepatocellular carcinoma | HCC | 29 |
| Journal of Clinical Oncology | Finn 2020 | Pembrolizumab As Second-Line Therapy in Patients With Advanced Hepatocellular Carcinoma in KEYNOTE-240: A Randomized, Double-Blind, Phase III Trial | PD1 | Pembrolizumab | Hepatocellular carcinoma | HCC | 279 |
| Lancet Oncol | Zhu 2018 | Pembrolizumab in patients with advanced hepatocellular carcinoma previously treated with sorafenib (KEYNOTE-224): a non-randomised, open-label phase 2 trial | PD1 | Pembrolizumab | Hepatocellular carcinoma | HCC | 104 |
| New England Jourl of Medicine | Ferris 2016 | Nivolumab for recurrent squamous-cell carcinoma of the head and neck | PD1 | Nivolumab | Head and neck cancer | HNSCC | 236 |
| Journal of Clinical Oncology | Bauml 2017 | Pembrolizumab for platinum- and cetuximab-refractory head and neck cancer: Results from a single-arm, phase II study | PD1 | Pembrolizumab | Head and neck cancer | HNSCC | 171 |
| Lancet | Burtness 2019 | Pembrolizumab alone or with chemotherapy versus cetuximab with chemotherapy for recurrent or metastatic squamous cell carcinoma of the head and neck (KEYNOTE-048): a randomised, open-label, phase 3 study | PD1 | Pembrolizumab | Head and neck cancer | HNSCC | 301 |
| Lancet | Cohen 2019 | Pembrolizumab versus methotrexate, docetaxel, or cetuximab for recurrent or metastatic head-and-neck squamous cell carcinoma (KEYNOTE-040): a randomised, open-label, phase 3 study | PD1 | Pembrolizumab | Head and neck cancer | HNSCC | 246 |
| European Journal of Cancer | Segal 2019 | Safety and efficacy of durvalumab in patients with head and neck squamous cell carcinoma: results from a phase I/II expansion cohort | PDL1 | Durvalumab | Head and neck cancer | HNSCC | 62 |
| European Journal of Cancer | Zandberg 2019 | Durvalumab for recurrent or metastatic head and neck squamous cell carcinoma: Results from a single-arm, phase II study in patients with °›25% tumour cell PD-L1 expression who have progressed on platinum-based chemotherapy | PDL1 | Durvalumab | Head and neck cancer | HNSCC | 112 |
| JAMA Oncol | Siu 2019 | Safety and Efficacy of Durvalumab With or Without Tremelimumab in Patients With PD-L1-Low/Negative Recurrent or Metastatic HNSCC: The Phase 2 CONDOR Randomized Clinical Trial | PDL1 | Durvalumab | Head and neck cancer | HNSCC | 65 |
| Annals of Oncology | Ferris 2020 | Durvalumab with or without tremelimumab in patients with recurrent or metastatic head and neck squamous cell carcinoma: EAGLE, a randomized, open-label phase III study | PDL1 | Durvalumab | Head and neck cancer | HNSCC | 237 |
| JAMA Oncology | Rizvi 2020 | Durvalumab with or without Tremelimumab vs Standard Chemotherapy in First-line Treatment of Metastatic Non-Small Cell Lung Cancer: The MYSTIC Phase 3 Randomized Clinical Trial | PDL1 | Durvalumab | Head and neck cancer | HNSCC | 369 |
| N Engl J Med | Motzer 2015 | Nivolumab versus Everolimus in Advanced Rel-Cell Carcinoma | PD1 | Nivolumab | Genitourinary cancer | Kidney | 406 |
| Cancer Sci | Yamazaki 2017 | Cytokine biomarkers to predict antitumor responses to nivolumab suggested in a phase 2 study for advanced melanoma | PD1 | Nivolumab | Melanoma | melanoma | 35 |
| Cancer Science | Yamazaki 2019 | Long-term follow up of nivolumab in previously untreated Japanese patients with advanced or recurrent malignt melanoma | PD1 | Nivolumab | Melanoma | melanoma | 24 |
| Journal of Clinical Oncology | Larkin 2018 | Overall Survival in Patients With Advanced Melanoma Who Received Nivolumab Versus Investigator's Choice Chemotherapy in CheckMate 037: A Randomized, Controlled, Open-Label Phase III Trial | PD1 | Nivolumab | Melanoma | melanoma | 268 |
| N Engl J Med | Robert 2015 | Nivolumab in previously untreated melanoma without BRAF mutation | PD1 | Nivolumab | Melanoma | Melanoma | 210 |
| European Journal of Cancer | Nathan 2019 | Safety and efficacy of nivolumab in patients with rare melanoma subtypes who progressed on or after ipilimumab treatment: a single-arm, open-label, phase II study (CheckMate 172) | PD1 | Nivolumab | Melanoma | melanoma | 1008 |
| N Engl J Med | Weber 2017 | Adjuvant Nivolumab versus Ipilimumab in Resected Stage III or IV Melanoma | PD1 | Nivolumab | Melanoma | melanoma | 452 |
| N Engl J Med | Wolchok 2017 | Overall Survival with Combined Nivolumab and Ipilimumab in Advanced Melanoma | PD1 | Nivolumab | Melanoma | melanoma | 313 |
| Lancet Oncol | Long 2018 | Combition nivolumab and ipilimumab or nivolumab alone in melanoma brain metastases: a multicentre randomised phase 2 study | PD1 | Nivolumab | Melanoma | melanoma | 43 |
| N Engl J Med | Eggermont 2018 | Adjuvant Pembrolizumab versus Placebo in Resected Stage III Melanoma | PD1 | Pembrolizumab | Melanoma | melanoma | 509 |
| European Journal of Cancer | Hamid 2017 | Fil alysis of a randomised trial comparing pembrolizumab versus investigator-choice chemotherapy for ipilimumab-refractory advanced melanoma | PD1 | Pembrolizumab | Melanoma | melanoma | 357 |
| Journal of Clinical Oncology | Kluger 2019 | Long-term survival of patients with melanoma with active brain metastases treated with pembrolizumab on a phase II trial | PD1 | Pembrolizumab | Melanoma | melanoma | 23 |
| The Lancet Oncology | Long 2019 | Epacadostat plus pembrolizumab versus placebo plus pembrolizumab in patients with unresectable or metastatic melanoma (ECHO-301/KEYNOTE-252): a phase 3, randomised, double-blind study | PD1 | Pembrolizumab | Melanoma | melanoma | 352 |
| N Engl J Med | Robert 2015 | Pembrolizumab versus Ipilimumab in Advanced Melanoma | PD1 | Pembrolizumab | Melanoma | melanoma | 555 |
| Journal of Clinical Oncology | Nghiem 2019 | Durable tumor regression and overall survival in patients with advanced Merkel cell carcinoma receiving pembrolizumab as first-line therapy | PD1 | Pembrolizumab | Mixed cancer types | Merkel Cell Carcinoma | 50 |
| N Engl J Med | Nghiem 2016 | PD-1 Blockade with Pembrolizumab in Advanced Merkel-Cell Carcinoma | PD1 | Pembrolizumab | Mixed cancer types | Merkel Cell Carcinoma | 26 |
| The Lancet Oncology | Kaufman 2016 | Avelumab in patients with chemotherapy-refractory metastatic Merkel cell carcinoma: a multicentre, single-group, open-label, phase 2 trial | PDL1 | Avelumab | Mixed cancer types | Merkel cell carcinoma | 88 |
| Clinical Cancer Research | Okada 2019 | Clinical efficacy and safety of nivolumab: Results of a multicenter, open-label, single-arm, Japanese phase II study in malignt pleural mesothelioma (MERIT) | PD1 | Nivolumab | Mixed cancer types | mesothelioma | 34 |
| Lancet Oncology | Scherpereel 2019 | Nivolumab or nivolumab plus ipilimumab in patients with relapsed malignt pleural mesothelioma (IFCT-1501 MAPS2): a multicentre, open-label, randomised, non-comparative, phase 2 trial | PD1 | Nivolumab | Mixed cancer types | mesothelioma | 63 |
| Intertiol Jourl of Clinical Oncology | Nomura 2020 | Multicenter prospective phase II trial of nivolumab in patients with unresectable or metastatic mucosal melanoma | PD1 | Nivolumab | Melanoma | mucosal melanoma | 20 |
| British Journal of Cancer | Vijayvergia 2020 | Pembrolizumab monotherapy in patients with previously treated metastatic high-grade neuroendocrine neoplasms: joint alysis of two prospective, non-randomised trials | PD1 | Pembrolizumab | Mixed cancer types | Neuroendocrine Neoplasms | 29 |
| Clinical cancer research | Strosberg 2020 | Efficacy and Safety of Pembrolizumab in Previously Treated Advanced Neuroendocrine Tumors: Results From the Phase 2 KEYNOTE-158 Study | PD1 | Pembrolizumab | Mixed cancer types | Neuroendocrine Tumors | 107 |
| J Clin Oncol | Ma 2018 | Antitumor Activity of Nivolumab in Recurrent and Metastatic sopharyngeal Carcinoma: An Intertiol, Multicenter Study of the Mayo Clinic Phase 2 Consortium (NCI-9742) | PD1 | Nivolumab | Head and neck cancer | NPC | 45 |
| Esmo Open | Nishio 2017 | Multicentre phase II study of nivolumab in Japanese patients with advanced or recurrent non-squamous non-small cell lung cancer | PD1 | Nivolumab | Lung cancer | NSCLC | 76 |
| Cancer Science | Hida 2017 | Efficacy and safety of nivolumab in Japanese patients with advanced or recurrent squamous non-small cell lung cancer | PD1 | Nivolumab | Lung cancer | NSCLC | 35 |
| N Engl J Med | Brahmer 2015 | Nivolumab versus Docetaxel in Advanced Squamous-Cell Non-Small-Cell Lung Cancer | PD1 | Nivolumab | Lung cancer | NSCLC | 131 |
| N Engl J Med | Borghaei 2015 | Nivolumab versus Docetaxel in Advanced Nonsquamous Non-Small-Cell Lung Cancer | PD1 | Nivolumab | Lung cancer | NSCLC | 292 |
| Lancet Oncol | Rizvi 2015 | Activity and safety of nivolumab, an anti-PD-1 immune checkpoint inhibitor, for patients with advanced, refractory squamous non-small-cell lung cancer (CheckMate 063): a phase 2, single-arm trial | PD1 | Nivolumab | Lung cancer | NSCLC | 117 |
| New England Jourl of Medicine | Carbone 2017 | First-line nivolumab in stage IV or recurrent non-small-cell lung cancer | PD1 | Nivolumab | Lung cancer | NSCLC | 267 |
| Lung Cancer | Lee 2018 | Nivolumab in advanced non-small-cell lung cancer patients who failed prior platinum-based chemotherapy | PD1 | Nivolumab | Lung cancer | NSCLC | 100 |
| European Journal of Cancer | Felip 2020 | CheckMate 171: A phase 2 trial of nivolumab in patients with previously treated advanced squamous non-small cell lung cancer, including ECOG PS 2 and elderly populations | PD1 | Nivolumab | Lung cancer | NSCLC | 811 |
| Journal of Thoracic Oncology | Wu 2019 | Nivolumab Versus Docetaxel in a Predomintly Chinese Patient Population With Previously Treated Advanced NSCLC: CheckMate 078 Randomized Phase III Clinical Trial | PD1 | Nivolumab | Lung cancer | NSCLC | 337 |
| N Engl J Med | Hellmann 2019 | Nivolumab plus Ipilimumab in Advanced Non-Small-Cell Lung Cancer | PD1 | Nivolumab | Lung cancer | NSCLC | 396 |
| The Lancet Oncology | Goldberg 2020 | Pembrolizumab for magement of patients with NSCLC and brain metastases: long-term results and biomarker alysis from a non-randomised, open-label, phase 2 trial | PD1 | Pembrolizumab | Lung cancer | NSCLC | 42 |
| Lancet | Herbst 2016 | Pembrolizumab versus docetaxel for previously treated, PD-L1-positive, advanced non-small-cell lung cancer (KEYNOTE-010): a randomised controlled trial | PD1 | Pembrolizumab | Lung cancer | NSCLC | 339 |
| Lancet | Herbst 2016 | Pembrolizumab versus docetaxel for previously treated, PD-L1-positive, advanced non-small-cell lung cancer (KEYNOTE-010): a randomised controlled trial | PD1 | Pembrolizumab | Lung cancer | NSCLC | 343 |
| Eur J Cancer | Levy 2019 | Randomised phase 2 study of pembrolizumab plus CC-486 versus pembrolizumab plus placebo in patients with previously treated advanced non-small cell lung cancer | PD1 | Pembrolizumab | Lung cancer | NSCLC | 49 |
| The Lancet Respiratory Medicine | Middleton 2020 | Pembrolizumab in patients with non-small-cell lung cancer of performance status 2 (PePS2): a single arm, phase 2 trial | PD1 | Pembrolizumab | Lung cancer | NSCLC | 60 |
| Lancet | Mok 2019 | Pembrolizumab versus chemotherapy for previously untreated, PD-L1-expressing, locally advanced or metastatic non-small-cell lung cancer (KEYNOTE-042): a randomised, open-label, controlled, phase 3 trial | PD1 | Pembrolizumab | Lung cancer | NSCLC | 637 |
| Journal of Clinical Oncology | Reck 2019 | Updated alysis of KEYNOTE-024: Pembrolizumab versus platinum-based chemotherapy for advanced non®Csmall-cell lung cancer with PD-L1 tumor proportion score of 50% or greater | PD1 | Pembrolizumab | Lung cancer | NSCLC | 154 |
| JAMA Oncology | Theelen 2019 | Effect of Pembrolizumab after Stereotactic Body Radiotherapy vs Pembrolizumab Alone on Tumor Response in Patients with Advanced Non-Small Cell Lung Cancer: Results of the PEMBRO-RT Phase 2 Randomized Clinical Trial | PD1 | Pembrolizumab | Lung cancer | NSCLC | 36 |
| Lancet | Fehrenbacher 2016 | Atezolizumab versus docetaxel for patients with previously treated non-small-cell lung cancer (POPLAR): a multicentre, open-label, phase 2 randomised controlled trial | PDL1 | Atezolimumab | Lung cancer | NSCLC | 142 |
| Journal of Thoracic Oncology | Fehrenbacher 2018 | Updated Efficacy Analysis Including Secondary Population Results for OAK: A Randomized Phase III Study of Atezolizumab versus Docetaxel in Patients with Previously Treated Advanced Non®CSmall Cell Lung Cancer | PDL1 | Atezolimumab | Lung cancer | NSCLC | 609 |
| Lancet Oncology | Geoerger 2020 | Atezolizumab for children and young adults with previously treated solid tumours, non-Hodgkin lymphoma, and Hodgkin lymphoma (iMATRIX): a multicentre phase 1-2 study | PDL1 | Atezolimumab | Lung cancer | NSCLC | 87 |
| Journal of Clinical Oncology | Peters 2017 | Phase II Trial of Atezolizumab As First-Line or Subsequent Therapy for Patients With Programmed Death-Ligand 1-Selected Advanced Non-Small-Cell Lung Cancer (BIRCH) | PDL1 | Atezolimumab | Lung cancer | NSCLC | 659 |
| Journal of Thoracic Oncology | Spigel 2018 | FIR: Efficacy, Safety, and Biomarker Analysis of a Phase II Open-Label Study of Atezolizumab in PD-L1-Selected Patients With NSCLC | PDL1 | Atezolimumab | Lung cancer | NSCLC | 137 |
| Lancet Oncol | Barlesi 2018 | Avelumab versus docetaxel in patients with platinum-treated advanced non-small-cell lung cancer (JAVELIN Lung 200): an open-label, randomised, phase 3 study | PDL1 | Avelumab | Lung cancer | NSCLC | 396 |
| Journal of Thoracic Oncology | Autonia 2019 | Clinical Activity, Tolerability, and Long-Term Follow-Up of Durvalumab in Patients With Advanced NSCLC | PDL1 | Durvalumab | Lung cancer | NSCLC | 304 |
| N Engl J Med | Antonia 2017 | Durvalumab after Chemoradiotherapy in Stage III Non-Small-Cell Lung Cancer | PDL1 | Durvalumab | Lung cancer | NSCLC | 473 |
| Annals of Oncology | Planchard 2020 | ARCTIC: durvalumab with or without tremelimumab as third-line or later treatment of metastatic non-small-cell lung cancer | PDL1 | Durvalumab | Lung cancer | NSCLC | 179 |
| Journal of Clinical Oncology | Zamarin 2020 | Randomized Phase II Trial of Nivolumab Versus Nivolumab and Ipilimumab for Recurrent or Persistent Ovarian Cancer: An NRG Oncology Study | PD1 | Nivolumab | Genitourinary cancer | ovarian | 49 |
| Journal of Clinical Oncology | Hamanishi 2015 | Safety and antitumor activity of Anti-PD-1 antibody, nivolumab, in patients with platinum-resistant ovarian cancer | PD1 | Nivolumab | Genitourinary cancer | Ovarian cancer | 20 |
| Anls of Oncology | Matulonis 2019 | Antitumor activity and safety of pembrolizumab in patients with advanced recurrent ovarian cancer: results from the phase II KEYNOTE-100 study | PD1 | Pembrolizumab | Genitourinary cancer | ovarian cancer | 376 |
| Gynecologic Oncology | Varga 2019 | Pembrolizumab in patients with programmed death ligand 1®Cpositive advanced ovarian cancer: Alysis of KEYNOTE-028 | PD1 | Pembrolizumab | Genitourinary cancer | ovarian cancer | 26 |
| JAMA Oncology | O'Reilly 2019 | Durvalumab with or Without Tremelimumab for Patients with Metastatic Pancreatic Ductal Adenocarcinoma: A Phase 2 Randomized Clinical Trial | PDL1 | Durvalumab | Gastrointestinal cancer | Pancrea | 32 |
| Journal for immunotherapy of cancer | Naing 2020 | Phase 2 study of pembrolizumab in patients with advanced rare cancers | PD1 | Pembrolizumab | Mixed cancer types | rare cancer | 127 |
| Journal of Clinical Oncology | Motzer 2015 | Nivolumab for metastatic rel cell carcinoma: Results of a randomized phase II trial | PD1 | Nivolumab | Genitourinary cancer | Rel cell carcinoma | 168 |
| Nature Medicine | McDermott 2018 | Clinical activity and molecular correlates of response to atezolizumab alone or in combination with bevacizumab versus sunitinib in renal cell carcinoma | PDL1 | Atezolimumab | Genitourinary cancer | renal cell carcinoma | 103 |
| Lancet Oncol | D’Angelo 2018 | Nivolumab with or without ipilimumab treatment for metastatic sarcoma (Alliance A091401): two open-label, non-comparative, randomised, phase 2 trials | PD1 | Nivolumab | Mixed cancer types | Sarcoma | 42 |
| Lancet Oncol | Tawbi 2017 | Pembrolizumab in advanced soft-tissue sarcoma and bone sarcoma (SARC028): a multicentre, two-cohort, single-arm, open-label, phase 2 trial | PD1 | Pembrolizumab | Mixed cancer types | sarcoma | 82 |
| Lancet Oncol | Antonia 2016 | Nivolumab alone and nivolumab plus ipilimumab in recurrent small-cell lung cancer (CheckMate 032): a multicentre, open-label, phase 1/2 trial | PD1 | Nivolumab | Lung cancer | SCLC | 98 |
| JAMA Oncology | Bauml 2019 | Pembrolizumab after Completion of Locally Ablative Therapy for Oligometastatic Non-Small Cell Lung Cancer: A Phase 2 Trial | PD1 | Pembrolizumab | Lung cancer | SCLC | 45 |
| Journal of Thoracic Oncology | Gadgeel 2018 | Phase II Study of Maintence Pembrolizumab in Patients with Extensive-Stage Small Cell Lung Cancer (SCLC) | PD1 | Pembrolizumab | Lung cancer | SCLC | 45 |
| Journal of Thoracic Oncology | Pujol 2019 | A Randomized Non-Comparative Phase II Study of Anti-Programmed Cell Death-Ligand 1 Atezolizumab or Chemotherapy as Second-Line Therapy in Patients With Small Cell Lung Cancer: Results From the IFCT-1603 Trial | PDL1 | Atezolimumab | Lung cancer | Small Cell Lung Cancer | 48 |
| The Lancet Oncology | Geoerger 2020 | Pembrolizumab in paediatric patients with advanced melanoma or a PD-L1-positive, advanced, relapsed, or refractory solid tumour or lymphoma (KEYNOTE-051): interim alysis of an open-label, single-arm, phase 1®C2 trial | PD1 | Pembrolizumab | Mixed cancer types | solid tumor or lymphoma | 142 |
| The Lancet Oncology | Davis 2020 | Nivolumab in children and young adults with relapsed or refractory solid tumours or lymphoma (ADVL1412): a multicentre, open-label, single-arm, phase 1®C2 trial | PD1 | Nivolumab | Mixed cancer types | solid tumors | 85 |
| Gastric Cancer | Bang 2019 | Pembrolizumab alone or in combition with chemotherapy as first-line therapy for patients with advanced gastric or gastroesophageal junction adenocarcinoma: results from the phase II nonrandomized KEYNOTE-059 study | PD1 | Pembrolizumab | Gastrointestinal cancer | Stomach | 31 |
| JAMA Oncol | Fuchs 2018 | Safety and Efficacy of Pembrolizumab Monotherapy in Patients With Previously Treated Advanced Gastric and Gastroesophageal Junction Cancer: Phase 2 Clinical KEYNOTE-059 Trial | PD1 | Pembrolizumab | Gastrointestinal cancer | Stomach | 259 |
| Annals of Oncology | Bang 2018 | Phase III, randomised trial of avelumab versus physician's choice of chemotherapy as third-line treatment of patients with advanced gastric or gastro-oesophageal junction cancer: Primary analysis of JAVELIN Gastric 300 | PDL1 | Avelumab | Gastrointestinal cancer | Stomach | 184 |
| Lancet Oncol | Giaccone 2018 | Pembrolizumab in patients with thymic carcinoma: a single-arm, single-centre, phase 2 study | PD1 | Pembrolizumab | Mixed cancer types | thymic carcinoma | 40 |
| Lancet Oncol | Balar 2017 | First-line pembrolizumab in cisplatin-ineligible patients with locally advanced and unresectable or metastatic urothelial cancer (KEYNOTE-052): a multicentre, single-arm, phase 2 study | PD1 | Pembrolizumab | Genitourinary cancer | urothelial cancer | 370 |
| Journal of Clinical Oncology | Galsky 2020 | Randomized Double-Blind Phase II Study of Maintence Pembrolizumab Versus Placebo After First-Line Chemotherapy in Patients With Metastatic Urothelial Cancer | PD1 | Pembrolizumab | Genitourinary cancer | urothelial cancer | 55 |
| N Engl J Med | Bellmunt 2017 | Pembrolizumab as Second-Line Therapy for Advanced Urothelial Carcinoma | PD1 | Pembrolizumab | Genitourinary cancer | Urothelial carcinoma | 270 |
| Lancet | Balar 2017 | Atezolizumab as first-line treatment in cisplatin-ineligible patients with locally advanced and metastatic urothelial carcinoma: a single-arm, multicentre, phase 2 trial | PDL1 | Atezolimumab | Genitourinary cancer | urothelial carcinoma | 119 |
| European Urology | Pal 2018 | Atezolizumab in Platinum-treated Locally Advanced or Metastatic Urothelial Carcinoma: Clinical Experience from an Expanded Access Study in the United States | PDL1 | Atezolimumab | Genitourinary cancer | urothelial carcinoma | 214 |
| Lancet | Powles 2018 | Atezolizumab versus chemotherapy in patients with platinum-treated locally advanced or metastatic urothelial carcinoma (IMvigor211): a multicentre, open-label, phase 3 randomised controlled trial | PDL1 | Atezolimumab | Genitourinary cancer | urothelial carcinoma | 459 |
| Nature Medicine | Powles 2019 | Clinical efficacy and biomarker analysis of neoadjuvant atezolizumab in operable urothelial carcinoma in the ABACUS trial | PDL1 | Atezolimumab | Genitourinary cancer | urothelial carcinoma | 95 |
| Lancet | Rosenberg 2016 | Atezolizumab in patients with locally advanced and metastatic urothelial carcinoma who have progressed following treatment with platinum-based chemotherapy: a single-arm, multicentre, phase 2 trial | PDL1 | Atezolimumab | Genitourinary cancer | urothelial carcinoma | 310 |
| European Urology | Sternberg 2019 | Primary Results from SAUL, a Multinational Single-arm Safety Study of Atezolizumab Therapy for Locally Advanced or Metastatic Urothelial or Nonurothelial Carcinoma of the Urinary Tract | PDL1 | Atezolimumab | Genitourinary cancer | urothelial carcinoma | 997 |
| Cancer Sci | Tamura 2019 | NA | PD1 | Nivolumab | Genitourinary cancer | uterine | 64 |
